# Intravenous ferric derisomaltose for iron-deficiency anemia associated with gastrointestinal diseases: a single-arm, randomized, uncontrolled, open-label study

**DOI:** 10.1007/s12185-022-03420-x

**Published:** 2022-07-22

**Authors:** Hiroshi Kawabata, Takeshi Tamura, Soichiro Tamai, Tomoki Takahashi, Jun Kato, Study Group, Ito Hiroaki, Tanaka Hironori, Hosokawa Takanori, Kitsukawa Yoshio, Ogata Shinichi, Yoshida Rihito, Iwase Shigeru, Kido Osamu, Akiho Hirotada, Kusumoto Hirotake, Matsuda Tomoki, Takahashi Shuji, Matsuyama Kiichi, Hamahata Yukihiro, Abe Hisanori

**Affiliations:** 1 Department of Hematology, National Hospital Organization Kyoto Medical Center, 1-1 Fukakusa Mukaihata-cho, Fushimi-ku, Kyoto, 612-8555 Japan; 2 Clinical Development Department, Nippon Shinyaku Co., Ltd, Kyoto, Japan; 3 Data Science Department, Nippon Shinyaku Co., Ltd, Kyoto, Japan; 4 Department of Gastroenterology, Graduate School of Medicine, Chiba University, Chiba, Japan

**Keywords:** Ferric derisomaltose, Iron-deficiency anemia, Intravenous iron preparation, Gastrointestinal disease

## Abstract

Iron-deficiency anemia (IDA) associated with gastrointestinal diseases is the second most common etiology of IDA in Japan, and is most often caused by gastrointestinal bleeding. A multicenter, single-arm (2 groups), open-label, phase III study was conducted to assess the efficacy and safety of ferric derisomaltose (FDI) when administered by intravenous (IV) bolus injection (*n* = 30) or drip infusion (*n* = 10) in Japanese patients with IDA associated with gastrointestinal diseases. The primary endpoint, which was the mean maximum change in hemoglobin (Hb) concentration from baseline, was 4.33 (95% confidence interval, 3.82–4.83) g/dL in the overall population (4.27 [3.83–4.71] g/dL in the bolus injection group and 4.49 [2.69–6.29] g/dL in the drip infusion group). Treatment-emergent adverse events (TEAEs) were reported in 24 patients (60.0%) in the overall population (18 patients [60.0%] in the bolus injection group and 6 patients [60.0%] in the drip infusion group). No serious treatment-related TEAEs or unexpected safety findings were reported during the study. These findings reveal a favorable efficacy and safety profile for FDI when administered by IV bolus injection or drip infusion in Japanese patients with IDA associated with gastrointestinal diseases.

**Supplementary Information:**

The online version contains supplementary material available at 10.1007/s12185-022-03420-x.

## Introduction

Iron-deficiency anemia (IDA) is the most common anemia, affecting more than 1 billion individuals [1]. The most common cause of IDA in Japan is menorrhagia; however, in men and postmenopausal women, it is most often caused by gastrointestinal bleeding [2]. Guideline-based therapy for IDA includes treatment of its underlying cause and restoration of iron stores [3] through oral or intravenous (IV) routes. However, the standard treatment of IDA with oral iron has limitations in patients with inflammatory bowel disease (IBD), as it can cause adverse reactions. Furthermore, IV iron is more effective, shows a faster response, and is better tolerated than oral iron [4]. Therefore, IV iron should be considered as first-line treatment in patients with clinically active IBD and/or gastrointestinal bleeding who require immediate iron supplementation.

When this study was initiated, the only available IV iron preparation in Japan was saccharated ferric oxide (SFO), with the maximum daily dose as low as 120 mg. Guidelines recommend injection of 40–120 mg of iron on consecutive days until the cumulative dose is achieved [3]. However, SFO has non-negligible risks of hypophosphatemia and fibroblast growth factor 23 (FGF23)-related osteomalacia [5]. Ferric derisomaltose (FDI), also known as iron isomaltoside 1000 (Pharmacosmos A/S [Denmark]), is an IV iron preparation approved for the indication of iron-deficiency in Europe since 2009 and is currently marketed worldwide [6]. FDI contains oligosaccharides with a molecular weight of approximately 1000 Da and is a linear and unbranched structure consisting predominantly of 3–5 glucose units with low immunological potential. Owing to the slow release of iron, FDI is considered to have a favorable toxicity profile in IDA [7–9]. A rapid IV infusion of high-dose iron is possible because FDI has a high stability, suitable molecular weight, and low risk of free iron-related toxicities. In Europe, both the IV drip infusion and IV bolus injection have been approved as routes of FDI administration. In a Phase I study of Japanese patients with IDA, FDI administration using a single bolus injection or drip infusion was found to be well tolerated (ClinicalTrials.gov identifier: NCT03013439). We recently completed a Phase III study that evaluated the efficacy and safety of FDI in Japanese patients with IDA caused by menorrhagia (JapicCTI-No: JapicCTI-194573) [10]. Our findings revealed the efficacy and safety of an IV drip infusion of up to 1000 mg of FDI followed by a second administration of an IV bolus injection of up to 500 mg in Japanese patients. A previous study conducted by European and Indian groups showed efficacy and acceptable safety profiles of both the IV bolus injection and IV infusion of FDI for the treatment of IDA in patients with IBD [11]. However, the efficacy and safety of FDI for the treatment of IDA due to causes other than menorrhagia and the efficacy and safety of the IV bolus injection of FDI for the first administration have not yet been evaluated in Japanese patients.

Expanding upon earlier research, this study aimed to investigate the efficacy and safety of FDI in Japanese patients with IDA caused by gastrointestinal bleeding, the second most common cause of IDA in Japan, and the efficacy and safety of IV bolus injection of FDI for the first administration in Japanese patients.

## Materials and methods

### Study design

We conducted a multicenter, single-arm (2 groups), randomized, uncontrolled, open-label, Phase III study to assess the efficacy and safety of FDI administered by either IV bolus injection or drip infusion in patients with IDA associated with gastrointestinal diseases at 15 study sites in Japan from February 2019 to April 2020. The protocol and informed consent forms (ICFs) were approved by the Institutional Review Board (IRB) at each participating site according to Good Clinical Practice (GCP) guidelines. This study was registered in the Japic Clinical Trials Information as JapicCTI-194572 and conducted in accordance with the ethical principles of the Declaration of Helsinki (October 2013) and GCP, as well as the study protocol. Before any study procedure, the ICF was signed and personally dated both by the patient and by the person who conducted the informed consent discussion.

### Patients

This study was designed for a 3:1 randomization by minimization to either FDI by bolus injection or FDI by drip infusion. At least 40 patients were stratified by hemoglobin (Hb) concentration at the time of informed consent (screening visit) and body weight at Week 0, both of which are important clinical variables for total iron dose determination. The sample size was determined considering the fact that the efficacy and safety of FDI in patients with different diseases and ethnic backgrounds have already been examined in previous studies [11, 12]. Patients were considered to be eligible at the primary registration if they were Japanese; ≥ 18 years old; diagnosed with IDA with gastrointestinal bleeding due to gastrointestinal diseases; and had been intolerant to oral iron preparations for the previous 2 years, had failed to respond to oral iron preparations for ≥ 1 month, or were considered to require immediate iron supplementation by the site principal investigator (a sub investigator). Final eligibility criteria, determined at the screening visit, were Hb < 11.0 g/dL, serum ferritin < 100 ng/mL if C-reactive protein (CRP) exceeded the reference range or < 30 ng/mL if CRP was within the reference range, and a transferrin saturation (TSAT) < 20% per the European consensus on the diagnosis and management of iron deficiency and anemia in inflammatory bowel diseases [4]. The inclusion criterion was changed from “total iron-binding capacity (TIBC) ≥ 360 μg/dL” to “TSAT < 20%” in November 2019 because TIBC values decrease with nutritional status, increasing age, and inflammation [3], and the TSAT test was used instead of the TIBC test, based on the criteria of Study P-IBD-01, which was conducted outside Japan [11]. The main exclusion criteria were anemia due to causes other than iron deficiency, iron overload or defective iron utilization, acute or chronic infections, risk of increasing severity of hypersensitivity, pregnant or nursing women, and a history of allergy to a component of IV iron preparations. Patients who had received either an erythropoiesis-stimulating agent or a blood transfusion within 8 weeks prior to the primary registration date or IV iron preparations within 4 weeks prior to the primary registration date and/or those for whom the site principal investigator chose prioritization of surgical treatment were also excluded at the primary registration. Patients with estimated glomerular filtration rate (eGFR) < 30 mL/min/1.73 m^2^, decompensated cirrhosis or active hepatitis, or risk of hypophosphatemia were excluded at the screening visit (by Week 0).

### Interventions

**Fig. 1:**
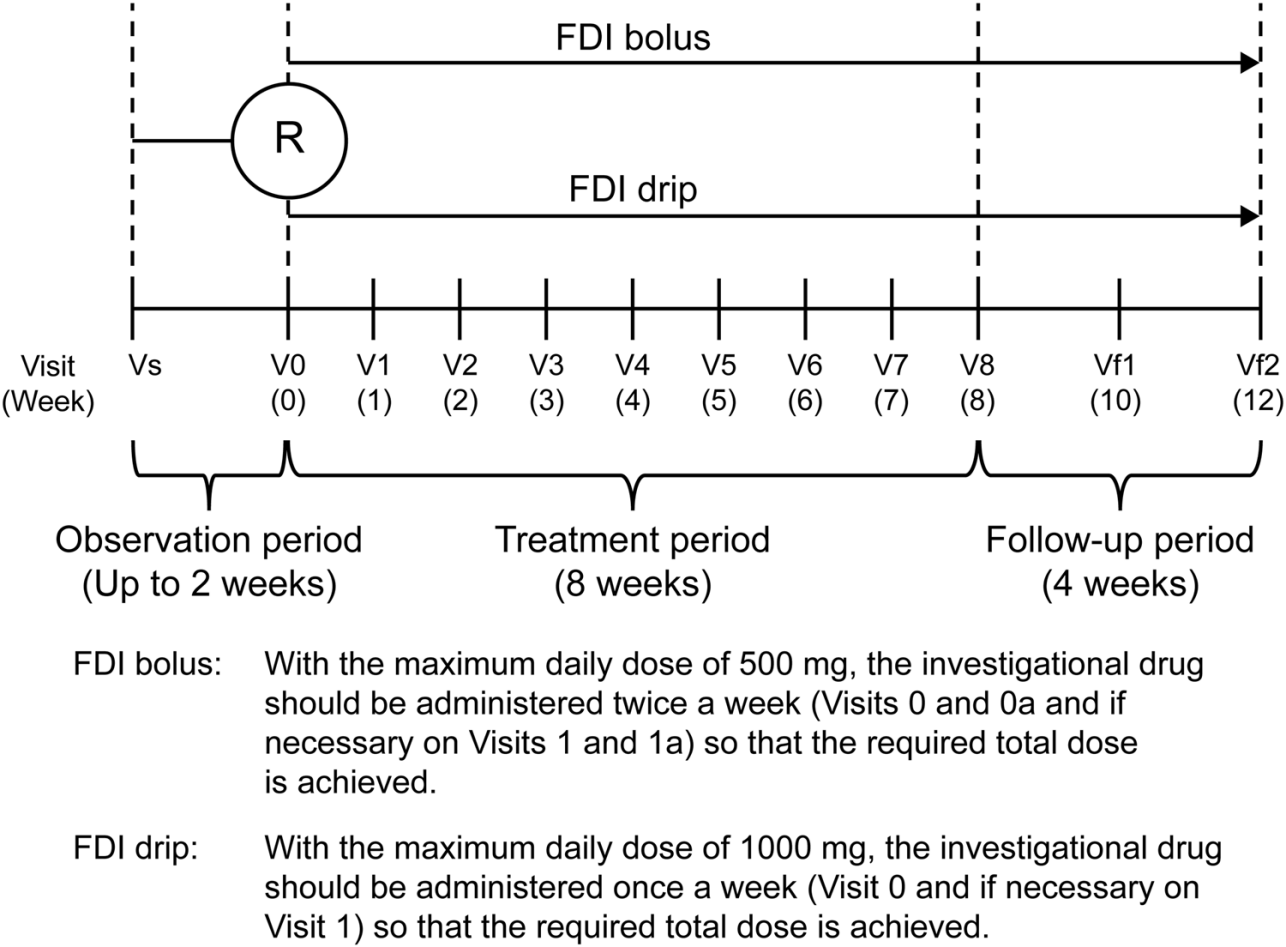
Study design in Japanese patients with iron-deficiency anemia associated with gastrointestinal diseases. *FDI* ferric derisomaltose, *R* randomization, *V* visit, *V0a* additional visit between V0 and V1, *V1a* additional visit between V1 and V2, *Vf1* follow-up visit 1, *Vf2* follow-up visit 2, *Vs* screening visit

The study consisted of 3 study periods: an observation period of up to 2 weeks, an 8-week treatment period with mandatory visits occurring once a week, and a 4-week follow-up period (Fig. 1). For both the FDI bolus injection group and the FDI drip infusion group, the total iron dose was determined using the Hb concentration and body weight according to a simplified table (Supplementary Table S1 in Online Resource 1). For patients weighing < 40 kg, the dose was calculated using the Uchida formula [13].

#### Bolus injection group dosing

For the bolus injection group, at Week 0 (Day 1), all patients received the first administration of FDI at a dose of 500 mg. The FDI bolus was either undiluted or diluted with physiological saline of ≤ 20 mL and administered over 2 min. The maximum daily dose was 500 mg. To administer the total iron dose, which was calculated individually for each patient, FDI was administered twice a week until the total dose was achieved.

#### Drip infusion group dosing

For the drip infusion group, at Week 0 (Day 1), all patients received the first administration of FDI diluted with physiological saline of up to 500 mL and administered over 15 min. For patients weighing ≥ 50 kg, the first dose was 1000 mg and for patients weighing < 50 kg, the FDI dose was calculated at 20 mg/kg of body weight. If the total iron dose was not fully administered at the first dose, the difference was administered at Week 1 (Days 8–10) as the second dose. If the second dose was ≤ 500 mg, it was administered as a bolus injection; if > 500 mg, it was administered by IV drip infusion.

#### Treatment interruption criteria

Treatment was interrupted if serum phosphorus decreased to < 2.0 mg/dL or if serum ferritin increased to > 500 ng/mL. Dosing was resumed if serum phosphorus increased to > 2.5 mg/dL and serum ferritin decreased to < 250 ng/mL.

### Endpoints

The primary efficacy endpoint was the maximum change in Hb concentration from baseline. Secondary endpoints were changes in iron-related biochemical parameters (i.e., Hb concentration, TSAT, and serum ferritin concentration) and cumulative iron dose achievement. The reticulocyte ratio was an additional efficacy endpoint. The safety endpoints were treatment-emergent adverse events (TEAEs), including symptoms of hypersensitivity, abnormalities in vital signs and physical examination, changes in laboratory parameters (i.e., hematology, serum chemistry, and urine tests), and abnormalities in standard 12-lead electrocardiography (ECG).

### Sample size determination

Considering the feasibility of recruitment, the target sample size was set to a minimum of 40 patients (30 patients for the bolus injection group and at least 10 patients for the drip infusion group). Assuming the effect size of this study was similar to that in the patient subgroup with gastrointestinal diseases in Study P-IDA-01 [12] (mean change in Hb = 2.62, standard deviation [SD] = 1.19), 30 patients provide ≥ 99.9% power to detect a significant change in Hb from baseline using a paired t-test with *α* = 0.05. For the safety assessment, a 10% incidence of TEAEs in 30 patients can be detected at a probability of ≥ 95%. A minimum of 10 patients were targeted for enrollment in the drip infusion group to obtain safety and efficacy data among IDA patients with diseases other than menorrhagia who received FDI through this route of administration.

### Statistical analysis

The full analysis set (FAS), defined as all patients who received ≥ 1 dose of FDI and had efficacy data available, was used for all efficacy analyses. Safety analyses were performed using the safety analysis set, defined as all patients who received ≥ 1 dose of FDI.

Baseline demographics were summarized using descriptive statistics. The mean (SD) was calculated for continuous variables, and the frequency and proportion were calculated for categorical variables. Dosing data are presented as descriptive statistics.

Descriptive statistics by treatment groups were calculated for the maximum change in Hb concentrations from baseline with 95% confidence intervals (CIs). Subgroup analyses by sex, age, the major primary disease of IDA, Hb concentrations at baseline, and eGFR at baseline were performed for the primary endpoint. For the Hb concentration, serum ferritin concentration, TSAT, and reticulocyte count, descriptive statistics were calculated for the value measured and the change from baseline at each analysis visit. For patients whose Hb had increased by ≥ 2 g/dL from baseline and patients with Hb ≥ 12.0 g/dL, the number and percentage of the patients reaching these laboratory thresholds at each analysis visit and over the treatment period were calculated. The number and percentage of patients who had achieved the cumulative total iron dose during the treatment period were also calculated. Adverse events were coded using the Medical Dictionary for Regulatory Activities (MedDRA), Version 22.0. TEAEs and treatment-related TEAEs were summarized overall and by treatment group. TEAEs and treatment-related TEAEs were summarized by subgroup (sex, age, the major primary disease of IDA, Hb concentration at baseline, and eGFR at baseline). For serum phosphorus, descriptive statistics were calculated for the value measured at each analysis visit and serum phosphorus grades were calculated as a post hoc analysis. All statistical analyses were performed using SAS^®^ software version 9.4 (SAS Institute, Cary, NC, USA). *P* values < 0.05 were considered statistically significant.

## Results

**Fig. 2:**
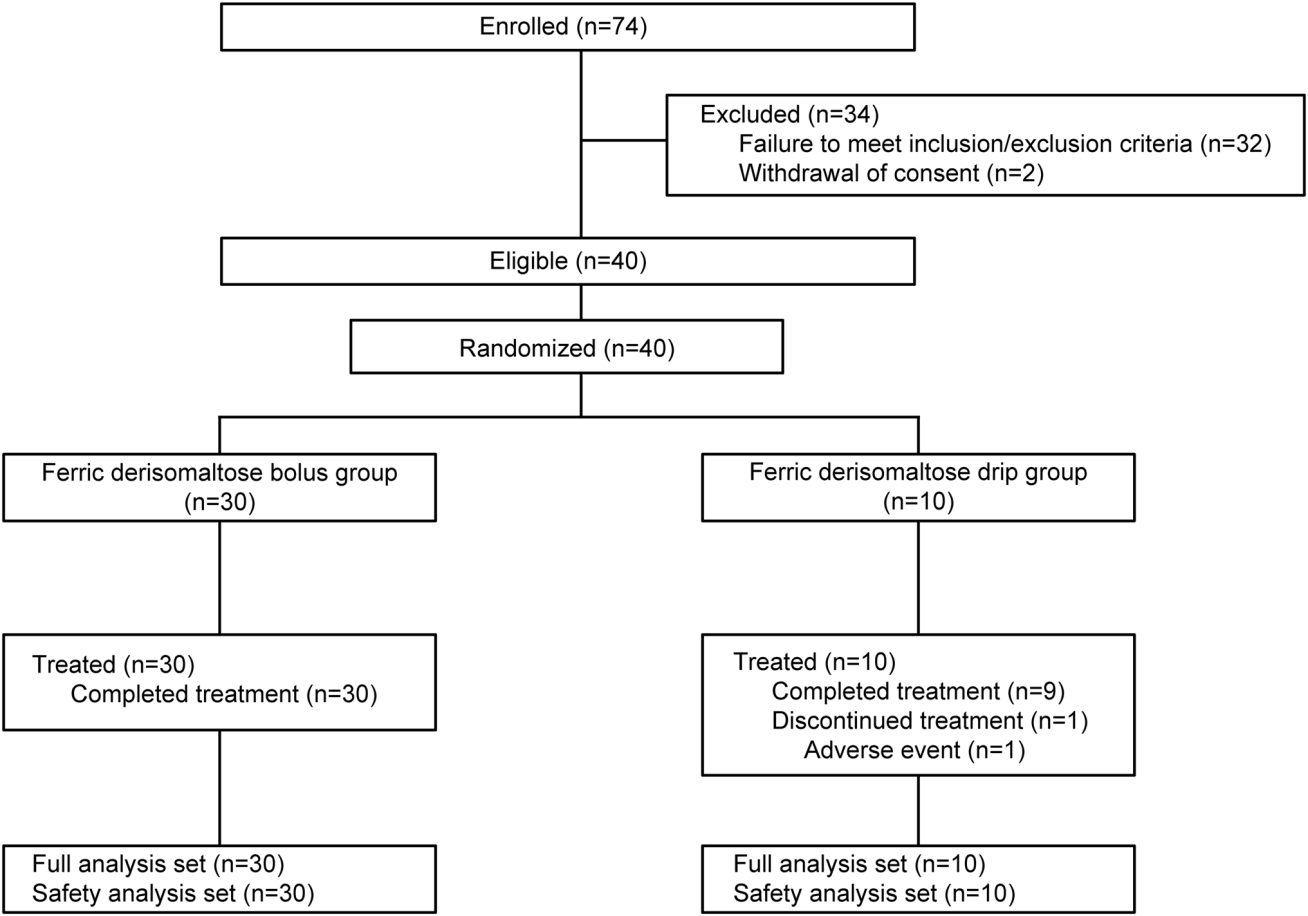
Patient disposition

A total of 74 patients were enrolled in the study at the primary registration. Of these, 34 were excluded at the screening visit. Forty patients were registered and randomized 3:1 to either the bolus injection group (30 patients) or the drip infusion group (10 patients). All patients received the study treatment. Of the 10 patients in the drip infusion group, 1 patient discontinued the study treatment due to a TEAE. All randomized patients completed the study. All 40 patients were included in the safety analysis set and FAS. The study disposition of all enrolled patients is presented in Fig. 2.

### Baseline characteristics

In the overall population, 47.5% were male, and the median age was 61.5 years. The most common primary disease was IBD (47.5%), followed by peptic ulcers (27.5%). The most common reason for IV iron administration was the need for immediate iron supplementation (87.5%). The mean ± SD Hb concentration at baseline was 9.43 ± 1.04 g/dL. Baseline characteristics were similar between the bolus injection and the drip infusion groups (Table 1).

**Table 1:** Demographics and baseline characteristics (full analysis set)

		FDI bolus (*N* = 30)	FDI drip (*N* = 10)	Overall (*N* = 40)
Age (years)	Mean (SD)	57.9 (18.8)	58.1 (15.5)	58.0 (17.8)
	Median	59.0	63.0	61.5
	Min–Max	27–89	32–75	27–89
	< 65	19 (63.3)	5 (50.0)	24 (60.0)
	≥ 65	11 (36.7)	5 (50.0)	16 (40.0)
Sex				
Male	*n* (%)	14 (46.7)	5 (50.0)	19 (47.5)
Female	*n* (%)	16 (53.3)	5 (50.0)	21 (52.5)
Weight (kg)	Mean (SD)	53.94 (11.47)	63.59 (13.94)	56.36 (12.67)
	Median	54.75	63.00	56.80
	Min–Max	28.1–78.9	47.6–96.8	28.1–96.8
	< 40	3 (10.0)	0 (0.0)	3 (7.5)
	40 to < 50	7 (23.3)	2 (20.0)	9 (22.5)
	50 to < 70	17 (56.7)	6 (60.0)	23 (57.5)
	≥ 70	3 (10.0)	2 (20.0)	5 (12.5)
Height (cm)	Mean (SD)	160.4 (8.5)	161.4 (6.1)	160.7 (7.9)
	Median	161.5	161.5	161.5
	Min–Max	137–180	154–170	137–180
BMI (kg/m^2^)	Mean (SD)	20.88 (3.68)	24.38 (5.12)	21.75 (4.30)
	Median	20.45	22.87	21.07
	Min–Max	10.2–29.3	20.1–37.3	10.2–37.3
Primary disease of IDA				
Inflammatory bowel disease (ulcerative colitis, Crohn's disease)	*n* (%)	15 (50.0)	4 (40.0)	19 (47.5)
Peptic ulcer (including gastric ulcer, duodenal ulcer, and NSAIDs ulcer)	*n* (%)	8 (26.7)	3 (30.0)	11 (27.5)
Other	*n* (%)	7 (23.3)	3 (30.0)	10 (25.0)
Reason for intravenous iron injection				
Intolerant to oral iron preparations within the previous 2 years	*n* (%)	2 (6.7)	2 (20.0)	4 (10.0)
Unresponsive to oral iron preparations for at least 1 month within the previous 2 years	*n* (%)	0 (0.0)	1 (10.0)	1 (2.5)
Prompt iron supplementation considered necessary	*n* (%)	28 (93.3)	7 (70.0)	35 (87.5)
Hemoglobin concentrations (g/dL) at baseline	Mean (SD)	9.41 (0.96)	9.46 (1.31)	9.43 (1.04)
	Median	9.35	9.20	9.25
	Min–Max	7.4–11.1	7.1–11.4	7.1–11.4
	< 8	2 (6.7)	1 (10.0)	3 (7.5)
	8 to < 10	18 (60.0)	6 (60.0)	24 (60.0)
	≥ 10	10 (33.3)	3 (30.0)	13 (32.5)
eGFR(mL/min/1.73 m^2^) at baseline	Mean (SD)	78.89 (21.92)	67.72 (20.52)	76.10 (21.87)
	Median	76.45	70.35	73.55
	Min–Max	31.3–123.4	33.7–106.2	31.3–123.4
	30 to < 60	5 (16.7)	3 (30.0)	8 (20.0)
	60 to < 90	17 (56.7)	6 (60.0)	23 (57.5)
	≥ 90	8 (26.7)	1 (10.0)	9 (22.5)

Percentages are based on *n*, the total number of patients in the treatment group at V0 for whom the information is available. The baseline value is the value measured before administration of the study treatment

*BMI* body mass index, *eGFR* estimated glomerular filtration rate, *FDI* ferric derisomaltose, *IDA* iron-deficiency anemia, *max* maximum, *min* minimum, *NSAID* nonsteroidal anti-inflammatory drug, *SD* standard deviation, *V0* Visit 0

### Exposure

Except for 1 patient in the FDI drip infusion group, all other patients received the study treatment as per the protocol. Of the overall population, 97.5% received the total iron dose during the 8-week treatment period (100.0% in the bolus injection group and 90.0% in the drip infusion group). The mean ± SD dose was 1253.3 ± 346.6 mg in the overall population (1237.7 ± 350.2 mg in the bolus injection group and 1300.0 ± 349.6 mg in the drip infusion group). The mean ± SD number of administrations was 2.57 ± 0.63 in the bolus injection group and 1.70 ± 0.48 in the drip infusion group.

### Endpoints

#### Efficacy

In the overall population, the mean ± SD maximum Hb concentration post baseline was 13.75 ± 1.30 g/dL. The mean maximum change in Hb concentration from baseline was 4.33 (95% CI, 3.82–4.83) g/dL in the overall population (4.27 [3.83–4.71] g/dL in the bolus injection group and 4.49 [2.69–6.29] g/dL in the drip infusion group) (Table 2). The analyses of the maximum change in Hb concentration from baseline for subgroups are presented in Supplementary Table S2 in Online Resource 1.

**Table 2:** Maximum change from baseline in hemoglobin concentrations (g/dL) (full analysis set)

	FDI bolus (*N* = 30)	FDI drip (*N* = 10)	Overall (*N* = 40)
Baseline			
Mean (SD)	9.41 (0.96)	9.46 (1.31)	9.43 (1.04)
Maximum value post-baseline			
Mean (SD)	13.68 (1.24)	13.95 (1.53)	13.75 (1.30)
Maximum change from baseline			
Mean (SD)	4.27 (1.18)	4.49 (2.52)	4.33 (1.58)
One-sample *t*-test	< 0.001	< 0.001	< 0.001
95% CI	3.83–4.71	2.69–6.29	3.82–4.83

The baseline value is the value measured before administration of the study drug

*n* = the total number of patients in the treatment group at V0 for whom the information is available

Maximum change was defined as the maximum value for change from baseline when measured from study day 2 to study day 91

*CI* confidence interval, *FDI* ferric derisomaltose, *SD* standard deviation, *V0* Visit 0

**Fig. 3:**
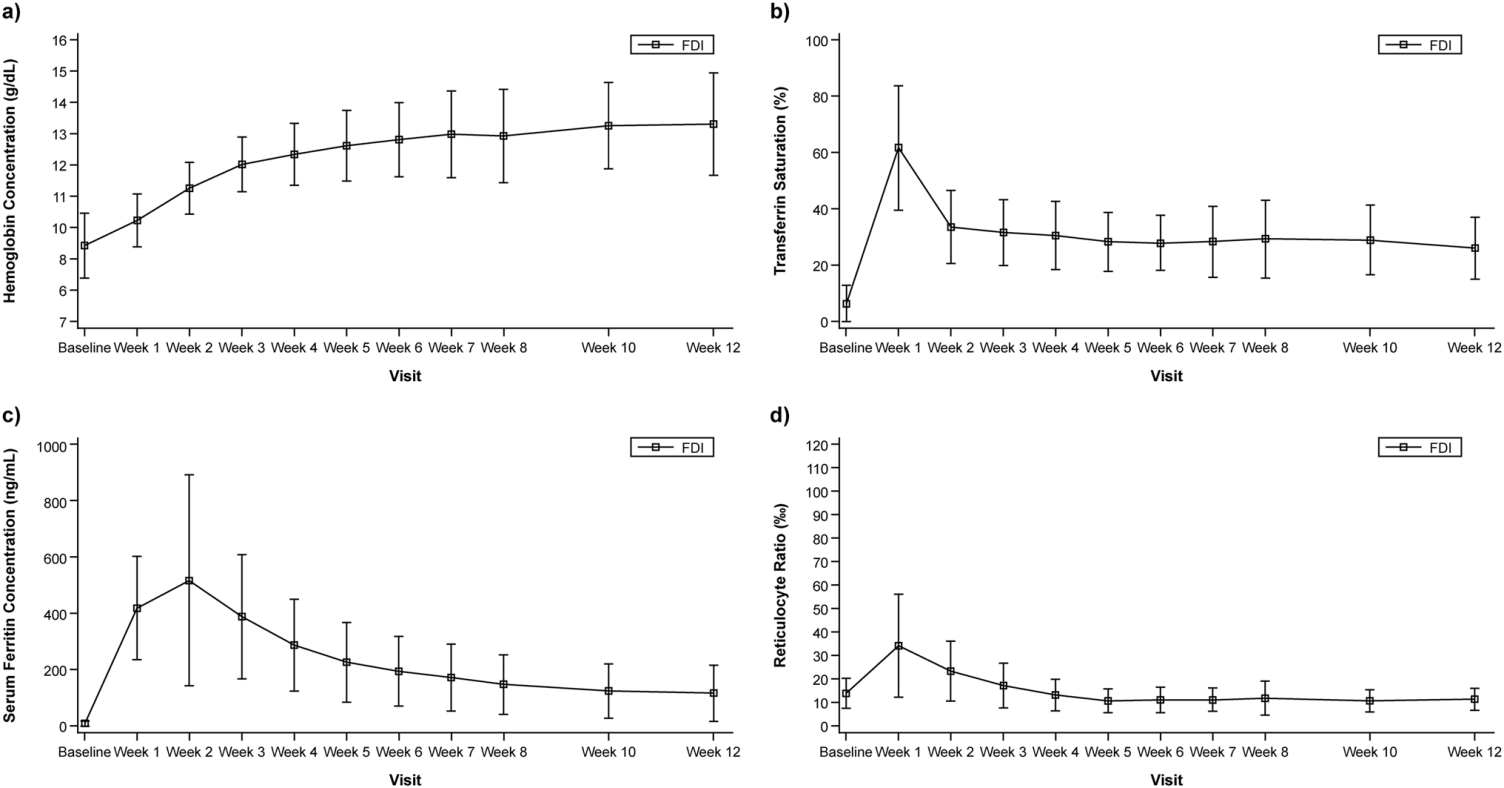
Laboratory value changes over the study period (mean ± SD) in (**a**) Hemoglobin concentration (g/dL), (**b**) Transferrin saturation (%), (**c**) Serum ferritin concentration (ng/mL), and (**d**) Reticulocyte ratio (‰). *FDI* ferric derisomaltose, *SD* standard deviation

In the overall population, the mean Hb concentration gradually increased until Week 12 and was maintained at > 12 g/dL after Week 3. Similar results were observed in each treatment group. The percentage of patients whose Hb concentration had increased from baseline by ≥ 2 g/dL increased over time up to Week 12, except for small decreases at Week 6 and Week 7. The percentage of patients with Hb concentration of ≥ 12.0 g/dL increased over time up to Week 12, except for small decreases at Week 6 and Week 8. Figure 3a presents Hb values at each study visit.

The mean ± SD TSAT value at baseline was 6.1 ± 6.4%. The maximum mean TSAT of 61.5% was observed at Week 1 in the overall population (67.3% in the bolus injection group and 35.3% in the drip infusion group) and then it gradually decreased. The mean TSAT values were maintained at > 20% during the follow-up period (Fig. 3b). Similar results were observed in each treatment group. The mean ± SD serum ferritin concentration at baseline was 8.83 ± 7.93 ng/mL in the overall population. The maximum serum ferritin concentration was observed at Week 2 (517.13 ng/mL) and then it gradually decreased to < 250 ng/mL after Week 5 (Fig. 3c). Similar results were observed in each treatment group. The mean ± SD reticulocyte ratio at baseline was 13.9 ± 6.5‰ in the overall population. It increased to 34.3‰ at Week 1, followed by a gradual decrease until Week 12 (Fig. 3d). Similar results were observed in each treatment group.

#### Safety

TEAEs were reported in 24 patients (60.0%) in the overall population (18 patients [60.0%] in the bolus injection group and 6 patients [60.0%] in the drip infusion group). Treatment-related TEAEs were reported in 12 patients (30.0%) in the overall population (9 patients [30.0%] in the bolus injection group and 3 patients [30.0%] in the drip infusion group). Table 3 summarizes TEAEs that occurred in ≥ 2 patients (≥ 5%) in the overall population. These included nasopharyngitis in seven patients (17.5%), hypophosphatemia in four patients (10.0%), pyrexia and urticaria in three patients (7.5%) each, and diarrhea, cystitis, headache, and hypertension in two patients (5.0%) each. Treatment-related TEAEs reported in ≥ 2 patients (≥ 5%) were as follows: hypophosphatemia in four patients (10.0%), urticaria in three patients (7.5%), and pyrexia in two patients (5.0%).

**Table 3:** Adverse events (safety analysis set)

Preferred term	TEAE			Treatment-related TEAE		
	FDI bolus (*N* = 30)	FDI drip (*N* = 10)	Overall (*N* = 40)	FDI bolus (*N* = 30)	FDI drip (*N* = 10)	Overall (*N* = 40)
Number of patients with at least one TEAE	18 (60.0)	6 (60.0)	24 (60.0)	9 (30.0)	3 (30.0)	12 (30.0)
Nasopharyngitis	7 (23.3)	0 (0.0)	7 (17.5)	0 (0.0)	0 (0.0)	0 (0.0)
Hypophosphatemia	3 (10.0)	1 (10.0)	4 (10.0)	3 (10.0)	1 (10.0)	4 (10.0)
Urticaria	1 (3.3)	2 (20.0)	3 (7.5)	1 (3.3)	2 (20.0)	3 (7.5)
Pyrexia	3 (10.0)	0 (0.0)	3 (7.5)	2 (6.7)	0 (0.0)	2 (5.0)
Diarrhea	2 (6.7)	0 (0.0)	2 (5.0)	1 (3.3)	0 (0.0)	1 (2.5)
Headache	2 (6.7)	0 (0.0)	2 (5.0)	1 (3.3)	0 (0.0)	1 (2.5)
Cystitis	2 (6.7)	0 (0.0)	2 (5.0)	0 (0.0)	0 (0.0)	0 (0.0)
Hypertension	0 (0.0)	2 (20.0)	2 (5.0)	0 (0.0)	0 (0.0)	0 (0.0)

Medical dictionary for regulatory activities version 22.0

Data are *n* (%)

TEAEs listed are those that occurred in ≥ 2 patients (≥ 5%) in the overall population

*FDI* ferric derisomaltose, *TEAE* treatment-emergent adverse event

The percentage of patients who met the treatment interruption criteria at least once in the treatment period was 62.5% in the overall population (66.7% in the bolus injection group and 50.0% in the drip infusion group). However, FDI administration was not interrupted for any patient because all these TEAEs occurred after the completion of FDI administration.

No TEAEs leading to death were reported. Serious TEAEs were reported in 3 patients (7.5%), including diverticular hemorrhage, ileus, and gastric cancer (1 patient each). No serious treatment-related TEAEs were reported. One patient had a severe TEAE of diverticular hemorrhage, which was not treatment related. In the drip infusion group, 1 patient had urticaria leading to drug withdrawal, which was mild and resolved with medication. One patient in the bolus injection group had urticaria leading to drug interruption, which was mild and resolved without medication.

**Fig. 4:**
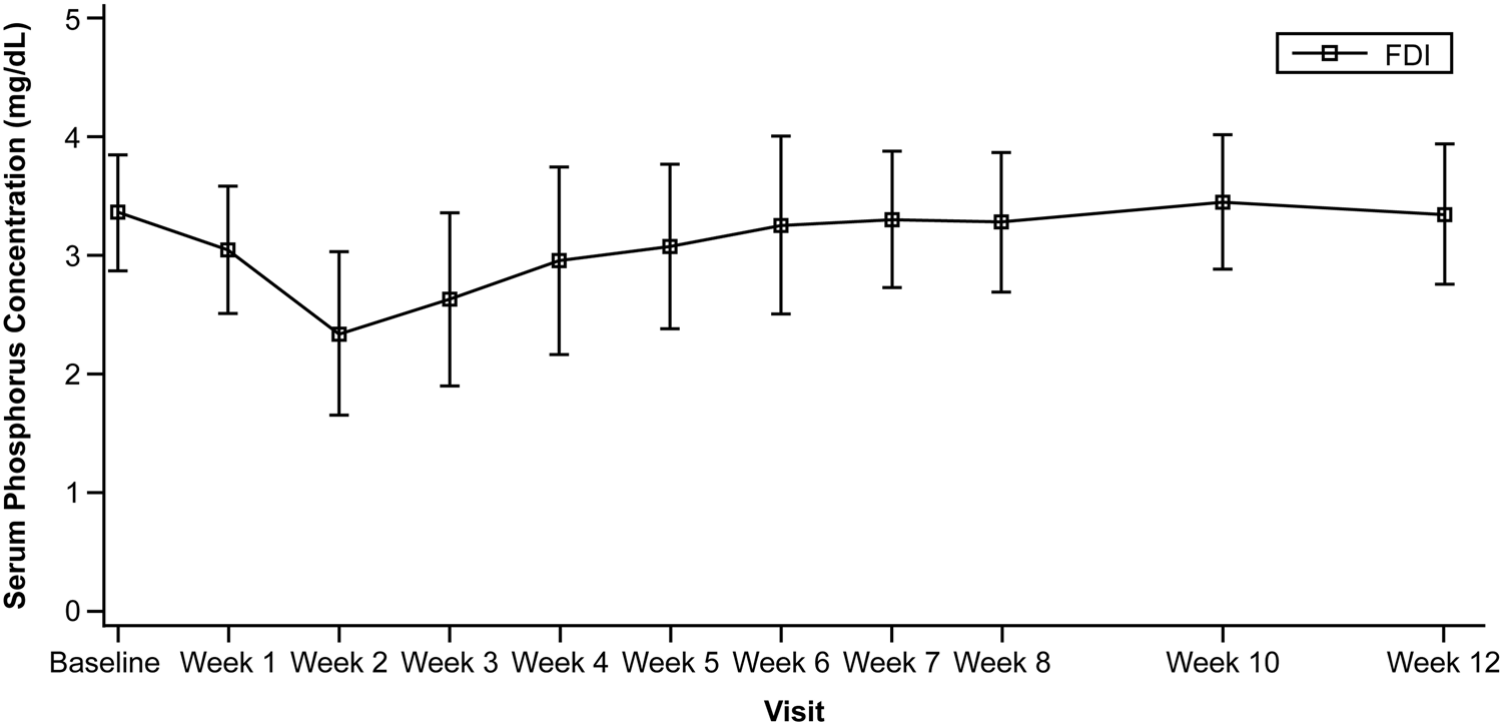
Laboratory value changes over the study period (mean ± SD) in serum phosphorus concentration (mg/dL). *FDI* ferric derisomaltose, *SD* standard deviation

The mean ± SD serum phosphorus value was 3.36 ± 0.49 mg/dL at baseline, and it was maintained ≥ 2.5 mg/dL, except at Week 2 (Fig. 4). Overall, 35.0% of patients had serum phosphorus levels < 2.0 mg/dL; however, none had serum phosphorus levels < 1.0 mg/dL (Supplementary Table S3 in Online Resource 1).

TEAEs by subgroup are presented in Supplementary Table S4 in Online Resource 1. Subgroup incidence of TEAEs and treatment-related TEAEs was similar, except when categorized by age and sex. Patients aged < 65 years had a higher incidence (70.8% and 37.5%, respectively) than those aged ≥ 65 years (43.8% and 18.8%, respectively). Similarly, the incidence of both TEAEs and treatment-related TEAEs was higher in female patients (76.2% and 38.1%, respectively) than in male patients (42.1% and 21.1%, respectively). Treatment-related TEAEs that were observed more frequently in female patients than in male patients were urticaria (14.3% vs. 0%) and pyrexia (9.5% vs. 0%).

## Discussion

A significant increase in Hb concentration from baseline, the primary endpoint, was observed in patients with IDA associated with gastrointestinal diseases in the overall population. Similar efficacy results were observed in both the FDI bolus injection and drip infusion groups. Furthermore, the results of the secondary endpoints supported these FDI efficacy results. The safety results from this study demonstrated an acceptable safety profile for FDI, including the safety of the IV bolus injection for the first administration in Japanese patients.

The findings of this study are consistent with the results of a previous study of Japanese premenopausal women with IDA associated with menorrhagia (JapicCTI-No: JapicCTI-194573) [10], which compared the efficacy and safety of IV administration of FDI with those of SFO. The least-squares mean for the maximum change in Hb concentration from baseline in the FDI group was 4.33 g/dL, which was noninferior to that in the SFO group. In the previous study, the incidence of TEAEs in the FDI group was 66.2%; the most common treatment-related TEAEs were pyrexia (8.4%), urticaria (8.0%), hypophosphatemia (5.9%), and serum ferritin increase (5.5%). Notably, the incidence of a serum phosphorus level < 2.0 mg/dL was significantly lower in the FDI group (8.4%) than in the SFO group (83.2%); severe hypophosphatemia (≤ 1.0 mg/dL) occurred in 6.7% of the SFO-treated patients compared with none in the FDI group.

The current study evaluated the efficacy and safety of IDA in postmenopausal women and men of all ages in Japan, including patients with moderate renal impairment. The analyses of the maximum change in Hb concentrations by subgroup showed consistent increases in Hb concentrations from baseline in all subgroups (Supplementary Table S2 in Online Resource 1). The mean maximum Hb concentrations were 14.14 g/dL in male patients and 13.40 g/dL in female patients, which were within the normal limits of Hb concentration, suggesting consistent efficacy regardless of sex. The maximum change in Hb concentration from baseline was comparable in patients across all ages. Similarly, the maximum change in Hb concentration from baseline was similar among patients with normal renal function and those with mild or moderate renal impairment.

Regarding the safety endpoint, in the current study, the incidence of TEAEs in both groups was similar to that in our previous study in patients with IDA associated with menorrhagia (JapicCTI-No: JapicCTI-194573) [10], and no serious treatment-related TEAEs were reported. However, a transient increase in the mean serum ferritin levels was observed during Week 1 through Week 4. Although serum ferritin value is widely used as an index for the amount of iron stored in the body, it is also affected by inflammation. In our cohort, a patient developed acute prostatitis with CRP elevation and showed a remarkably increased ferritin value (2370 ng/mL) at Week 2. Because the sample size was relatively small, this outlier strongly affected the results. Also, the ferritin levels immediately after the administration of IV iron may reflect rapid incorporation of iron into macrophages and not the amount of stored iron in the body. Notably, the mean serum ferritin level returned to the normal range after Week 4. Thus, we believe that the transient increase in serum ferritin observed in this study does not reflect systemic iron overload.

Incidence of TEAEs and treatment-related TEAEs was similar among subgroups, except for a higher incidence in patients aged < 65 years than in those aged ≥ 65 years and in female patients than in male patients; the incidence of urticaria and pyrexia was more frequent in female patients than in male patients, although the reason for this is unknown. Notably, there was no increasing trend in the incidence of TEAEs in patients aged ≥ 65 years, i.e., the elderly population, and in patients with eGFR 30 to < 60 mL/min/1.73 m^2^, i.e., the moderate renal impairment population. Thus, the subgroup analyses revealed no clinically relevant effect of demographic characteristics and comorbid diseases on the safety of FDI.

In two recent clinical trials of ferric carboxymaltose (FCM), another IV iron preparation that was recently approved in Japan, the lowest serum phosphorus levels (mean ± SD) were 1.45 ± 0.50 mg/dL and 1.57 ± 0.48 mg/dL, and 11 patients (9.3%) and 2 patients (5.3%) experienced severe hypophosphatemia (serum phosphorus levels < 1.0 mg/dL), respectively [14, 15]. Compared with patients treated with FCM in these studies, patients treated with FDI in the current study had better lowest values of mean serum phosphorus and no incidence of severe hypophosphatemia. Two other randomized controlled studies also demonstrated significantly lower rates of hypophosphatemia in patients treated with FDI compared with those treated with FCM [8, 16]. Therefore, the Medicines and Healthcare Products Regulatory Agency of the United Kingdom issued a recommendation in 2020 to monitor serum phosphate levels in patients with pre-existing risk factors for hypophosphatemia (such as low baseline phosphate, malnutrition, malabsorption, vitamin D deficiency, and hyperparathyroidism) and in those receiving long-term or multiple high-dose infusions of FCM but not FDI [17].

This study has at least two limitations—uncontrolled study design and a relatively small sample size. Despite these limitations, the safety and efficacy results were notably consistent with those from prior research. In a previous study, we observed that the efficacy of FDI was noninferior to that of SFO in Japanese patients with menorrhagia-associated IDA (JapicCTI-No: JapicCTI-194573) [10]. In addition, clinical trials outside of Japan have also reported consistent results on the safety and efficacy of FDI in patients with IDAs caused by various etiologies, including chronic kidney disease, IBD, chronic heart failure, cancer chemotherapy, and postpartum hemorrhage [18], confirming the efficacy and safety of iron supplementation by FDI administration through both IV bolus injection and drip infusion.

In conclusion, this study revealed a favorable efficacy and safety profile for the IV administration of FDI in the treatment of Japanese patients with IDA associated with gastrointestinal diseases. In addition, an acceptable safety of the IV bolus injection of FDI for the first administration in Japanese patients was also shown.

## Supplementary Information

Below is the link to the electronic supplementary material.

Supplementary file1 (DOCX 28 KB)
