# Supplementary material for: Intravenous ferric derisomaltose for iron-deficiency anemia associated with gastrointestinal diseases: a single-arm, randomized, uncontrolled, open-label study
Source: Int J Hematol. 2022 Jul 22;116(6):846–55. doi: 10.1007/s12185-022-03420-x (PMC9668782; doi:10.1007/s12185-022-03420-x)
Supplement: Supplementary file 1 — Supplementary file1 (DOCX 28 KB) [file 12185_2022_3420_MOESM1_ESM.docx]

**Electronic Supplementary Material**

***International Journal of Hematology***

**Intravenous ferric derisomaltose for iron deficiency anemia associated with gastrointestinal diseases: A single-arm, randomized, uncontrolled, open-label study**

**Author Names and Affiliations**

Hiroshi Kawabata^1^, Takeshi Tamura^2^, Soichiro Tamai^3^, Tomoki Takahashi^2^, Jun Kato^4^, and Study Group^5^

^1^ Department of Hematology, National Hospital Organization Kyoto Medical Center, Kyoto, Japan

^2^ Clinical Development Department, Nippon Shinyaku Co., Ltd., Kyoto, Japan

^3^ Data Science Department, Nippon Shinyaku Co., Ltd., Kyoto, Japan

^4^ Department of Gastroenterology, Graduate School of Medicine, Chiba University., Chiba, Japan

^5^ See Online Resource 1

**Corresponding Author**:

Hiroshi Kawabata, MD, PhD

Email: hkawabat@kuhp.kyoto-u.ac.jp

**Study Group**

Ito Hiroaki, Kinshukai Infusion Clinic

Tanaka Hironori, Takarazuka City Hospital

Hosokawa Takanori, Showa General Hospital

Kitsukawa Yoshio, Chiba Aoba Municipal Hospital

Ogata Shinichi, Saga-Ken Medical Centre Koseikan

Yoshida Rihito, Hirakata Kosai Hospital

Iwase Shigeru, Fujisawa City Hospital

Kido Osamu, Iwate Prefectural Central Hospital

Akiho Hirotada, Kitakyushu Municipal Medical Center

Kusumoto Hirotake, Nanpuh Hospital

Matsuda Tomoki, Sendai Kousei Hospital

Takahashi Shuji, Koseikai Takeda Hospital

Matsuyama Kiichi, Ijinkai Takeda General Hospital

Hamahata Yukihiro, Kokikai Tsujinaka Hospital Kashiwanoha

Abe Hisanori, Abe Digestive Endoscopy Clinic

These affiliations are at the time of the conduct of the study.

**Supplementary Table S1** Simplified table

| Pre-dose  Hb concentration (g/dL) | Weight  <40 kg | Weight  40 to <50 kg | Weight  50 to <70 kg | Weight  ≥70 kg |
| --- | --- | --- | --- | --- |
| ≥10 | Uchida formula^*^ | 750 mg | 1000 mg | 1500 mg |
| <10 | Uchida formula^*^ | 1000 mg | 1500 mg | 2000 mg |

^*^Uchida formula: [2.2×(16-pre-dose patient Hb concentration [g/dL])+10]×body weight (kg)

(The total iron dose was rounded to integers).

*Hb* hemoglobin.

**Supplementary Table S2** Maximum change from baseline in hemoglobin concentrations (g/dL) by subgroup (full analysis set)

|  | N | Mean (SD) |
| --- | --- | --- |
| Sex |  |  |
| Male | 19 | 4.66 (1.84) |
| Female | 21 | 4.02 (1.28) |
| Age (years) |  |  |
| <65 | 24 | 4.12 (1.58) |
| ≥65 | 16 | 4.63 (1.58) |
| Primary disease of IDA |  |  |
| Inflammatory bowel disease (ulcerative colitis, Crohn's disease) | 19 | 3.65 (1.20) |
| Peptic ulcer (including gastric ulcer, duodenal ulcer, and NSAIDs ulcer) | 11 | 5.15 (1.69) |
| Other | 10 | 4.70 (1.69) |
| Hemoglobin concentration (g/dL) at baseline |  |  |
| <8 | 3 | 6.00 (2.61) |
| 8 to <10 | 24 | 4.63 (1.32) |
| ≥10 | 13 | 3.37 (1.33) |
| eGFR (mL/min/1.73 m^2^) at baseline |  |  |
| 30 to <60 | 8 | 4.16 (1.60) |
| 60 to <90 | 23 | 4.55 (1.73) |
| ≥90 | 9 | 3.89 (1.16) |

n = the total number of patients in the treatment group at V0 for whom the information is available.

Maximum change was defined as the maximum value for change from baseline when measured from study day 2 to study day 91.

*eGFR* estimated glomerular filtration rate, *IDA* iron deficiency anemia, *NSAID* nonsteroidal anti-inflammatory drug, *SD* standard deviation, *V0* Visit 0.

**Supplementary Table S3** Serum phosphorus grade over the study period (mg/dL)

|  | Overall | | | | |
| --- | --- | --- | --- | --- | --- |
|  | N | Grade 1  ≥2.5 | Grade 2  2.0 to <2.5 | Grade 3  1.0 to <2.0 | Grade 4  <1.0 |
| Baseline | 40 | 39 (97.5) | 1 (2.5) | 0 (0.0) | 0 (0.0) |
| Week 1 | 40 | 36 (90.0) | 4 (10.0) | 0 (0.0) | 0 (0.0) |
| Week 2 | 38 | 14 (36.8) | 12 (31.6) | 12 (31.6) | 0 (0.0) |
| Week 3 | 39 | 24 (61.5) | 9 (23.1) | 6 (15.4) | 0 (0.0) |
| Week 4 | 39 | 32 (82.1) | 4 (10.3) | 3 (7.7) | 0 (0.0) |
| Week 5 | 39 | 33 (84.6) | 3 (7.7) | 3 (7.7) | 0 (0.0) |
| Week 6 | 38 | 35 (92.1) | 2 (5.3) | 1 (2.6) | 0 (0.0) |
| Week 7 | 39 | 37 (94.9) | 1 (2.6) | 1 (2.6) | 0 (0.0) |
| Week 8 | 40 | 38 (95.0) | 1 (2.5) | 1 (2.5) | 0 (0.0) |
| Week 10 | 40 | 40 (100.0) | 0 (0.0) | 0 (0.0) | 0 (0.0) |
| Week 12 | 40 | 37 (92.5) | 3 (7.5) | 0 (0.0) | 0 (0.0) |
| During the treatment period* | 40 | 11 (27.5) | 15 (37.5) | 14 (35.0) | 0 (0.0) |

Data are n (%).

*Number of patients with at least one post-baseline assessment in the worst-grade category.

**Supplementary Table S4** Incidence of adverse events by subgroup (safety analysis set)

|  | N | TEAE | Treatment-related TEAE |
| --- | --- | --- | --- |
| Sex |  |  |  |
| Male | 19 | 8 (42.1) | 4 (21.1) |
| Female | 21 | 16 (76.2) | 8 (38.1) |
| Age (years) |  |  |  |
| <65 | 24 | 17 (70.8) | 9 (37.5) |
| ≥65 | 16 | 7 (43.8) | 3 (18.8) |
| Primary disease of IDA |  |  |  |
| Inflammatory bowel disease (ulcerative colitis, Crohn's disease) | 19 | 12 (63.2) | 7 (36.8) |
| Peptic ulcer (including gastric ulcer, duodenal ulcer, and NSAIDs ulcer) | 11 | 7 (63.6) | 2 (18.2) |
| Other | 10 | 5 (50.0) | 3 (30.0) |
| Hemoglobin concentrations (g/dL) at baseline |  |  |  |
| <8 | 3 | 2 (66.7) | 2 (66.7) |
| 8 to <10 | 24 | 15 (62.5) | 8 (33.3) |
| ≥10 | 13 | 7 (53.8) | 2 (15.4) |
| eGFR (mL/min/1.73 m^2^) at baseline |  |  |  |
| 30 to <60 | 8 | 5 (62.5) | 2 (25.0) |
| 60 to <90 | 23 | 14 (60.9) | 6 (26.1) |
| ≥90 | 9 | 5 (55.6) | 4 (44.4) |

Data are n (%).

*eGFR* estimated glomerular filtration rate, *IDA* iron deficiency anemia, *NSAID* nonsteroidal anti-inflammatory drug, *TEAE* treatment-emergent adverse event.
